# Inheritance patterns, challenges, and outcomes of fracture management in osteopetrosis patients. CASE series and review of pertinent literature

**DOI:** 10.1016/j.amsu.2018.10.038

**Published:** 2018-11-08

**Authors:** Obada Hasan, Aly Zaheer Pathan, Hammad Naqi, Talal Aqueel, Pervaiz Hashmi, Riaz Hussain Lakdawala

**Affiliations:** a Department of Surgery, Aga Khan University Hospital, Karachi, Pakistan; b Graduate MBBS, Aga Khan University, Karachi, Pakistan; c Section of Orthopedics, Department of Surgery, Aga Khan University Hospital, Karachi, Pakistan

**Keywords:** Osteopetrosis, Fracture, Outcome, Case series

## Abstract

**Background:**

Osteopetrosis (OP) is a group of rare inheritable genetic disorders which show increased bone radiodensity on radiography. As no cure exists, careful symptomatic treatment is the mainstay in management due to brittle bone and frequent complications. We would like to present a case series of OP patients, their management, a review of literature about this rare disease and its genetic and inheritance patterns.

**Materials and methods:**

Retrospective case series of 6 patients with OP seen at our institution from 2010 to January 2018. We searched PubMed and Google Scholar for articles using the following keywords: *Osteopetrosis, Radiology, Fracture* and *Management* to review literature.

**Cases presentation:**

We present 6 cases of OP each showing diverse history of frequent fractures and describe the challenges faced during management and the long-term follow-up results.

**Results:**

Abnormal osteoclast activity in OP results in defective bone resorption with patients having varied clinical presentations. Bones are brittle, increasing risk of fractures. Osteosynthesis is the recommended first-choice treatment for osteopetrotic fractures despite the risk of failure. Good preoperative planning is critical. Genetic studies showed multiple genes to be involved and varied patterns of inheritance in different types of OP. Conservative management could including varied therapies has also been proposed.

**Conclusion:**

With all-inclusive preoperative planning and careful postoperative care surgical treatment of fractures in OP is effective. The cases presented showed that plate osteosynthesis and intramedullary nailing are suitable options. Genetic factors and inheritance pattern should be discussed with patients.

## Highlights

• Complications are frequent due to brittle bone and decreased marrow cavity.
• Inheritance pattern affects presentation of osteopetrosis.
• Use of bone morphogenic protein prevents delayed healing of fractures.
• Osteosynthesis with careful planning is the preferred treatment of fractures.
• Incidence of osteopetrosis may be higher than reported in Pakistan.

## 1 Introduction

Osteopetrosis (OP) is a rare group of inheritable genetic disorders, characterized by increased radiodensity of bone on radiographic examination [1], initially described by German radiologist Albers-Schönberg in 1904 [2].Often referred to as ‘marble bone disease’, diagnosis of OP is primarily based on clinical and radiological findings [1].

Three variants of OP existing in humans are infantile-malignant autosomal recessive, intermediate autosomal recessive (AR) and autosomal dominant (AD), each having varied features as described in Table 1. Prognosis is determined by type and pattern of genes affected, with the infantile form linked to a poorer prognosis compared to intermediate or AD form [3]. Due to its rarity prevalence has not been found, however incidence for AR variety is estimated to be 1 in 250,000 births and 1 in 20,000 births for the AD variety [1]. OP has varied presentations with benign types only leading to increased risk of fractures and the most aggressive form resulting in death within months due to destruction of bone marrow [[4], [5], [6], [7], [8]].

As there is no cure, treatment is symptomatic with management of complications. Care is required in treating OP due to the brittle nature of the bone and frequent occurrence of secondary complications like delayed union, non-union and osteomyelitis [1]. Complications of operative treatment arise due to brittleness of bone and obliteration of marrow cavity. This subsequently impedes drilling and cutting, resulting in complications including hardware failure, periprosthetic fractures, delayed union, pseudarthrosis, refracture, risk of iatrogenic fracture and periprosthetic infection [9,10]. Lack of knowledge and inadequate perioperative planning increases the risk of such operative complications and improper fixation of fracture [9].

In this study, we present six cases of OP seen and managed at our institution from 2010 to January 2018. The cases are presented in Table 2 with an account of their fractures and procedures in Table 3.

**Table 1:** Characteristics of osteopetrosis.

Characteristic	Adult Onset	Intermediate	Infantile
Inheritance	Autosomal dominant	Autosomal recessive	Autosomal recessive
Prevalence	1 in 20,000	–	1 in 250,000
Main Complaints	Increased risk of fractures, infection, cranial nerve defects	Increased risk of fractures, infection	Bone marrow failure
Diagnosis	Diagnosed incidentally	–	Diagnosed early (<1year age)
Prognosis	Good	Poor	Poor

## 2 Methodology

This case series includes 6 patients seen at a tertiary care university hospital and level-1 trauma center from 2010 to January 2018 with their notes being reviewed retrospectively. Patients visited our hospital due to either failure of their previous treatment or getting another fracture. Past history was taken from the patient with emphasis on past surgical history if operated previously. They were diagnosed as having Osteopetrosis following multiple hospital visits with fractures and on radiological findings. All procedures were performed by three senior orthopedic consultants with experience of more than 10 years who are familiar in dealing with this disease. Patients underwent routine preoperative assessment. We ruled out presence of infection by detail history and clinical examination followed by preoperative blood markers (CRP and WBC count) and confirmed perioperatively by the absence of infected purulent fluid or necrotic tissue. And postoperatively none needed HDU or ICU and were shifted to the general ward for routine postoperative care. Approval from institutional ethics review committee was taken prior to start of the study. The research registry number for this study is researchregistry3724.

We searched PubMed and Google Scholar for articles using the following keywords: *Osteopetrosis, Radiology, Fracture*. The articles were then reviewed for information regarding OP including genetics and inheritance, clinical features, available treatments, complications and new treatments.

This work has been reported in line with the PROCESS criteria [11].

## 3 Results

Table 2 describes the six cases seen by us followed by Table 3 which reports procedures which took place for each case.

**Table 2:** Presentation of 6 cases.

Nactame	Age	Sex	Inheritance	Orthopedic Features	Non- Orthopedic Features	Family History
Case 1	55 years	Male	AD	Left thigh pain; Left femur oblique mid shaft fracture	None	Positive for OP
Case 2	39 years	Male	AR (intermediate variety)	Subtrochanteric fracture left femur; Hyper dense bones; Lack of medullary differentiation	None	Not known
Case 3	23 years	Male	AD	Right thigh pain and swelling; Right femur subtrochanteric fracture; thickened cortices; genu valgum bilateral lower limb	Hemophilia A; pallor	Father diagnosed with OP
Case 4	30 years	Male	AD	Right leg pain; Right tibia stress fracture; Varus deformity right tibia; valgus deformity left tibia, increased cortical thickening compared to previous radiographs; blade plate in right femur with broken distal screw	None	Brother, sister and 5 cousins diagnosed with OP and suffer similar problems
Case 5	3 Month 15 Days	Male	AR	Midshaft fracture of right clavicle; No intramedullary canal visible; Most bones show whited-out appearance	Tachypnea; Fever; Parathyroid hormone 283 pg/ml	Uncle has OP; 2 Aunts had OP; Parents had consanguineous marriage; Anemia; Deafness; Blindness
Case 6	58 years	Male	AD	Fracture of elbow (radius)	None	Son diagnosed with OP

**Table 3:** Procedure history, complications and outcomes.

Name; Age at fracture (years)	Pathology and Treatment	Complications	Management	Outcome
Case 1; 32	Right femur fracture; Plate osteosynthesis	Infection of implant	Removal of implant	Healed fracture but with broken implant remaining (Fig. 1A)
Case 1; 40	Left femur fracture; Plate osteosynthesis	Infection of implant	Removal of implant	Healed fracture with deformity (Fig. 1A)
Case 1; 55	Oblique fracture of midshaft of left femur; Plate osteosynthesis (Fig. 1B and C)	None	–	1 year 3-month follow-up demonstrated good bone alignment and healing (Fig. 1D)
Case 2; 24	Left femur fracture; Plate osteosynthesis (Fig. 1H)	Procedure at different facility	–	
Case 2; 32	Right femur fracture; Plate osteosynthesis (Fig. 1H)	Procedure at different facility	–	
Case 2; 39	Right tibia deformity; Osteotomy and intramedullary (IM) nailing (Fig. 1E–G)	None	–	Well healed on follow-up
Case 2; 40	Periprosthetic fracture of right femur; Removal of plate and IM nailing (Fig. 2A and B)	None	–	Well healed on 4-year follow-up (Fig. 2C)
Case 2; 43	Left tibia fracture; Osteotomy and IM nailing (Fig. 2D and E)	None	–	Well healed on 2-year follow-up (Fig. 2F)
Case 2; 45	Subtrochanteric fracture left femur; Removal of plate and IM nailing (Fig. 2G)	Drill bit broke during drilling of medullary canal (Fig. 2H)	Broken drill bit retrieved through lateral cortex	1 and a half-year follow-up showed healthy callus formation and good healing (Fig. 2I)
Case 3; 23	Subtrochanteric fracture right femur; Plate osteosynthesis	None	–	7-month follow-up showed satisfactory healing. Full weight bearing.
Case 4; 16	Left femur fracture; DHS	Infected implant;	Multiple debridement and antibiotics; implant removed	
Case 4; 21	Right femur fracture; Blade plate	Procedure at different facility	–	
Case 4; 23	Right tibia fracture; conservative treatment	Procedure at different facility	–	
Case 4; 24	Bilateral tibia fracture; conservative treatment	Procedure at different facility	–	
Case 4; 30	Right tibia fracture; advised removal of blade plate and IM nailing	Refused treatment	–	Lost to follow-up
Case 5; 3month	Fracture of midshaft of right clavicle; arm sling	None	–	4-month follow-up showed good healing
Case 5; 4month	Bone marrow transplant and on immunosuppressant	Pneumonia, electrolyte imbalance, gastroenteritis on multiple occasions	Treated as inpatient at our facility due to various complications	At 7-month follow-up had multiple admissions for complications
Case 6; 58years	Fracture of elbow (radius); arm sling	None	–	Good healing after 6-months

**Fig. 1:**
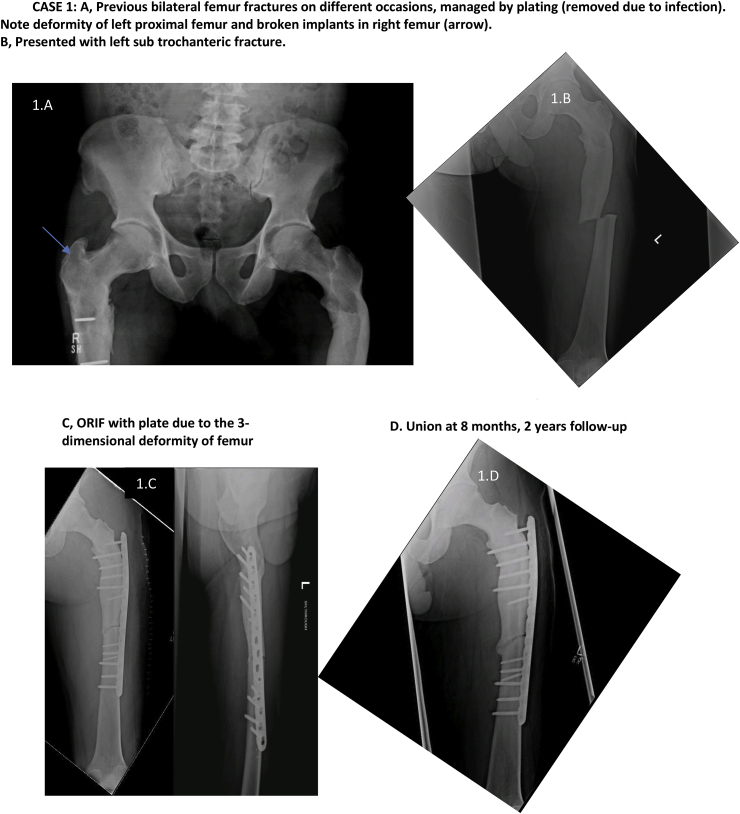

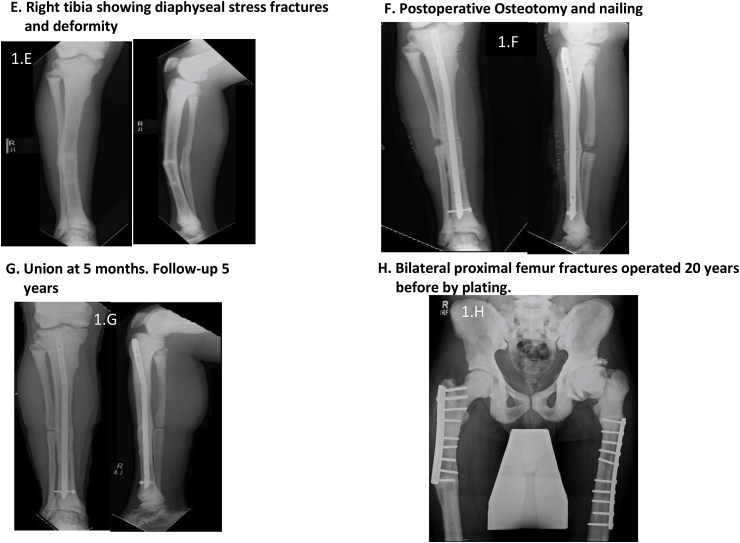
Radiographs of cases 1 and 2 (first three procedures).

**Fig. 2:**
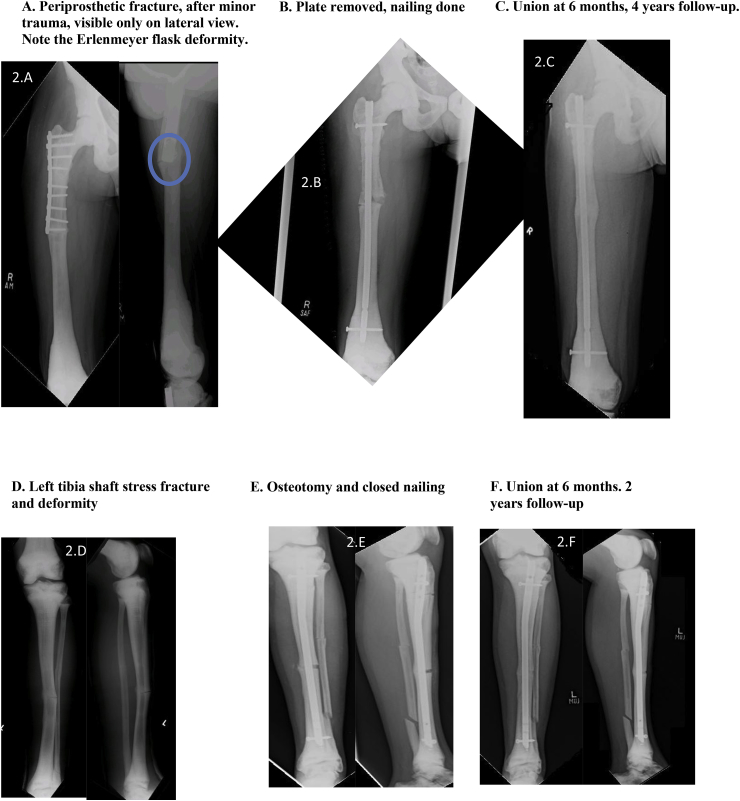

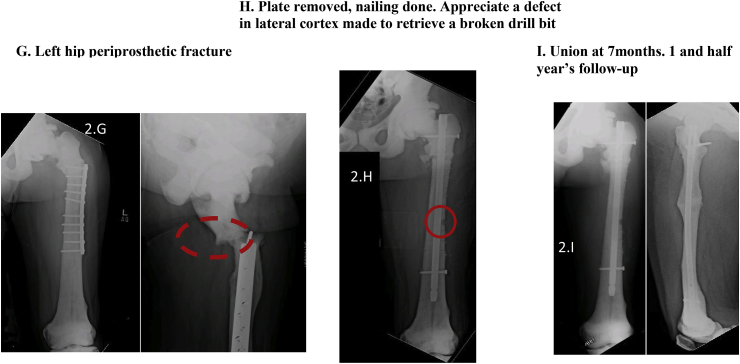
Radiographs of case 2 (next 3 procedures).

## 4 Discussion

### 4.1 Overview

Osteopetrosis is a rare group of genetic conditions with abnormal osteoclast activity resulting in ineffective or no bone resorption [5,12]. Patients with OP often suffer hematological abnormalities such as anemia and thrombocytopenia, for which investigation and perioperative correction is required. Patients with osteopetrosis can present with a variety of conditions such as back pain, bone pain, recurrent fractures, degenerative arthritis and infections [6,13]. In autosomal dominant OP (ADO), patients display characteristics such as thickened cranial vault, resulting in cranial nerve deficits, and delayed healing of fractures [[5], [6], [7]]. On radiography attributes such as sclerosis of skull, spine and pelvis, Erlenmeyer flask deformity (Fig. 2A), as well as ‘bone in bone’ appearance of vertebrae and phalanges is seen [1]. Whole body bone scintigraphy has been suggested as a method of diagnosis and assessment [14].

### 4.2 Literature review

Several case reports and small-scale case series have reported treatment of fractures in patients with osteopetrosis. Aslan et al. reported 2 cases of proximal femoral fracture treated with open reduction and internal fixation (ORIF) using cortical screws and anatomic plate for a subtrochanteric fracture in one case and only spongious screws in the second for a femoral neck fracture. No post-operative complications were noted in either case, however drill bits broke twice in one of the cases [5]. Golden et al. used a right angle Dynamic Compression Screw implant for a transverse fracture at level of lesser trochanter [12]. Amit et al. also reported fixation of subtrochanteric fractures of the femur with distal femoral locking compression plate on the contralateral side of fracture [15].

Zhang et al. described the treatment of hip osteoarthritis in an OP patient with total hip arthroplasty (THA). Patient was treated conservatively following a periprosthetic fracture. It was suggested that following THA conservative management of fracture was preferred due to decreased risk of complications [16]. Farfán et al. reported ORIF of oblique supracondylar fracture of the left humerus with simple, intra-articular, rotated fragment with capitellum involvement, and fracture in the base of the coronoid process. Recovery showed good range of motion and bone healing a year later. They recommended planning well preoperatively due to difficulties such as breaking of drill bits, bone overheating and difficult screw fixation [17]. Post-operatively it is suggested to closely care for the patient due to high risk of complications [17,18]. Table 4 provides a summary of challenges in managing OP.

**Table 4:** Difficulties faced in treatment of OP fractures.

	Difficulties	Suggested Resolution
1.	Broken/Bent drill bit	Multiple drill bits, diamond drill bits, use of staggered drill system
2.	Infection	Careful, intense post-operative care; inform patient of the risk
3.	Hard bone	Slow speed, high-torque electric drills; clearance of drill grooves
4.	Bone overheating	Frequent cooling with physiological saline; drilling pauses
5.	Periprosthetic fracture	Treat conservatively in older patients or if risk of complications high
6.	Slow healing of fracture	Use of Bone morphogenic protein graft to promote healthy callus formation
7.	Narrow and hard medullary canal	Drilling patiently and under fluoroscopy, use of manual drill to mark entry point

Studies recommend osteosynthesis as first-choice in the treatment of osteopetrotic fractures despite the risk of failure but good preoperative planning is crucial [5,16,19,20]. It is recommended to have multiple drill bits and screws, cooling with saline and low speed high-torque drill and also diamond drills [5].

### 4.3 Clinical presentation and surgical options

The first case, following previous procedures had an infection requiring removal of implant. Broken screws left. Fracture healed with deformity. Plate osteosynthesis was successful and despite radiographic healing at 15 months follow up, fracture line remained, corroborating with ADO. He presented having had multiple previous fractures causing tibial deformity. Records and radiographs from procedures at other hospitals were unavailable. Despite a drill bit breaking while nailing the left femur of the second patient, in comparison to other studies, no significant complication was noted, and adequate healing was observed post-operatively. Compared to the first patient the second patient had more frequent occurrence of fractures which may be related to them having different varieties of OP with different inheritance patterns causing the second patients’ bones being more prone to fracture or may be related to second patient being less careful after initial diagnosis. Our third patient presented with a proximal femur fracture despite minimal trauma and a deformity of lower limbs often seen in OP. He had satisfactory result following fixation. The fourth patient had a history of multiple fractures, lower limb deformities and a strong family history. Following the first procedure which resulted in postoperative infection of implant he had procedures at different facilities and the records of which were unobtainable. Our fifth patient had a clavicular fracture in infancy. Whited out appearance on radiograph and requirement for bone marrow transplant suggest lack of medullary cavity a feature more common in infantile OP. Strong family history of OP and cranial nerve defects and parents consanguineous marriage are likely in AR OP though cranial nerve defects are also seen in AD OP. Raised parathyroid hormone may suggest secondary hyperparathyroidism as a result of receptor resistance to the hormone [21].This patient continued to have a poor prognosis as is normally the case with infantile OP. Prognosis in this case can be compared to case 2 who survived to 45 years of age despite being initially diagnosed as having infantile OP. This makes it likely that case 2 had intermediate variety of OP. Our sixth case presented with radial fracture at elbow after a fall from ground level treated with an arm sling and had good healing of fracture at 6- months. All cases seen by us were male and in most cases inheritance pattern was AD.

### 4.4 Genetic counseling and inheritance

Modern genetic analysis allows gene identification in the disorder, however no specific gene defect was detected [4]. Due to the common origin of osteoclasts and macrophages, mutations in IKBKG, CalDAG-GEF1 and kindlin-3 are suspected to be involved in autosomal recessive OP variants with immune deficiency [1]. Cases described by Guerrini et al. and Sobacchi et al. show a relation between RANKL and RANK gene mutations and OP [22,23]. An error in the TCIRG1 gene, codes for osteoclast H+-ATPase pump, was identified in 60% of patients with severe infantile OP [24]. Chloride channel defects caused by dominant mutations of CLCN-7 result in ADO. CLCN-7 mutations are also seen in 15% of patients with severe autosomal recessive form and in some with intermediate OP [1,4]. Carbonic Anhydrase II (CAII) gene was the first error detected in OP patients. There was reduced activity of CAII enzyme which plays a role in bone resorption. Though the mutation was seen in less than 5% of patients with autosomal recessive OP, the role of CAII in the kidney may explain why patients have tubular acidosis [1,12].

### 4.5 Non-operative options

Some cases in literature report treating OP fractures conservatively with considerable outcome. Non-operative treatment options included hip Spica plaster cast, traction, splint and non-weight bearing [15,16]. Alternative treatments suggested include use of bone morphogenic proteins in place of autografts to promote callus formation during healing of fractures post-operatively [12]. Vitamin D3 has been suggested to increase bone resorption by stimulating osteoclasts. Erythropoietin may be used to correct anemia and gamma-interferon to delay disease progression and improve of white blood cell function. Allogenic bone marrow transplant may be used to reverse autosomal recessive form of OP [14]. Given the severity of infantile OP hematopoietic stem cell transplant (HSCT) has been suggested at an early age for patients. Only a few patients reported improvement in vision while most reported no further deterioration in vision [1].

## 5 Conclusion

Incidence of OP may be more than globally reported numbers for both AR and AD variations of the disease especially in Pakistan due to higher number of consanguineal marriages. Surgical treatment of fractures in osteopetrosis is effective with comprehensive pre-operative planning and vigilance for complications as seen by the literature review and our experience. The cases presented showed that plate osteosynthesis and intramedullary nailing are both suitable options however plate osteosynthesis is preferred due to difficulty and risk in drilling the narrow medullary cavity in the brittle bone of OP patients. Limitations in our study were unavailability of records of procedures at other hospitals. Case control or cross-sectional studies would be useful to measure the prevalence of this rare disease and generate theories for future intervention studies.

## Ethical approval

Ethical approval was taken from the Ethics Review Committee, Pakistan at the Aga Khan University prior to the start of the study.

The reference number for the study is 4450-Sur-ERC-16.

## Funding

No funding from any source.

## Author contribution

Obada Hasan-writing of manuscript.

Aly Zaheer Pathan-writing of manuscript.

Hammad Naqi-data collection, writing.

Talal Aqueel-data collection.

Pervaiz Hashmi-writing.

Riaz Hussain Lakdawala- Primary investigator, writing.

## Conflict of interest

No conflicts of interest.

## Research registration number

researchregistry3724.

## Guarantor

Obada Hasan.

Riaz Hussain Lakdawala.

Aly Zaheer Pathan.

Hammad Naqi.

Talal Aqueel.

Pervaiz Hashmi.

## Consent

Consent has been obtained from all patients involved in this study for publication of this case series and accompanying images.

## Provenance and peer review

Not commissioned, externally peer reviewed.

## Appendix A Supplementary data

Process Checklist Process Checklist
